# Election by Concours

**Published:** 1847-11

**Authors:** 


*Election by Concours.*—It is stated, that the three Faculties of Medicine, of Paris, Montpellier, and Strasbourg, have given their voices, in answer to the request of their opinion, made by the Minister of Public Instruction, in favour of the maintenance of the election to vacant professorships by concours. The French Reform Bill just passed the Chamber of Peers, annulled the mode of election by concours for professorships, which had existed for some years, and it is on this ground that most of the opposition to the Bill is raised by the French profession.—*Ibid.*

